# Supplementary material for: Swimming Activity Alleviates the Symptoms of Attention: Deficit Hyperactivity Disorder (ADHD) a Case Report
Source: Healthcare (Basel). 2023 Jul 11;11(14):1999. doi: 10.3390/healthcare11141999 (PMC10379488; doi:10.3390/healthcare11141999)
Supplement: Supplementary file 1 [file healthcare-11-01999-s001.zip › healthcare-2431493-supplementary.pdf]

Document S1. Beck's Depression Inventory

This depression inventory can be self-scored. The scoring scale is at the end of the questionnaire.

1.
- 0 I do not feel sad.
- 1 I feel sad
- 2 I am sad all the time and I can't snap out of it.
- 3 I am so sad and unhappy that I can't stand it.

2.
- 0 I am not particularly discouraged about the future.
- 1 I feel discouraged about the future.
- 2 I feel I have nothing to look forward to.
- 3 I feel the future is hopeless and that things cannot improve.

3.
- 0 I do not feel like a failure.
- 1 I feel I have failed more than the average person.
- 2 As I look back on my life, all I can see is a lot of failures.
- 3 I feel I am a complete failure as a person.

4.
- 0 I get as much satisfaction out of things as I used to.
- 1 I don't enjoy things the way I used to.
- 2 I don't get real satisfaction out of anything anymore.
- 3 I am dissatisfied or bored with everything.

5.
- 0 I don't feel particularly guilty
- 1 I feel guilty a good part of the time.
- 2 I feel quite guilty most of the time.
- 3 I feel guilty all of the time.

6.
- 0 I don't feel I am being punished.
- 1 I feel I may be punished.
- 2 I expect to be punished.
- 3 I feel I am being punished.

7.
- 0 I don't feel disappointed in myself.
- 1 I am disappointed in myself.
- 2 I am disgusted with myself.
- 3 I hate myself.

8.
- 0 I don't feel I am any worse than anybody else.
- 1 I am critical of myself for my weaknesses or mistakes.
- 2 I blame myself all the time for my faults.
- 3 I blame myself for everything bad that happens.

9.
- 0 I don't have any thoughts of killing myself.
- 1 I have thoughts of killing myself, but I would not carry them out.
- 2 I would like to kill myself.
- 3 I would kill myself if I had the chance.

10.
- 0 I don't cry any more than usual.
- 1 I cry more now than I used to.
- 2 I cry all the time now.
- 3 I used to be able to cry, but now I can't cry even though I want to.

11.
- 0 I am no more irritated by things than I ever was.
- 1 I am slightly more irritated now than usual.
- 2 I am quite annoyed or irritated a good deal of the time.
- 3 I feel irritated all the time.

12.
- 0 I have not lost interest in other people.
- 1 I am less interested in other people than I used to be.
- 2 I have lost most of my interest in other people.
- 3 I have lost all of my interest in other people.

13.
- 0 I make decisions about as well as I ever could.
- 1 I put off making decisions more than I used to.
- 2 I have greater difficulty in making decisions more than I used to.
- 3 I can't make decisions at all anymore.

14.
- 0 I don't feel that I look any worse than I used to.
- 1 I am worried that I am looking old or unattractive.
- 2 I feel there are permanent changes in my appearance that make me look unattractive
- 3 I believe that I look ugly.

15.
- 0 I can work about as well as before.
- 1 It takes an extra effort to get started at doing something.
- 2 I have to push myself very hard to do anything.
- 3 I can't do any work at all.

16.
- 0 I can sleep as well as usual.
- 1 I don't sleep as well as I used to.
- 2 I wake up 1-2 hours earlier than usual and find it hard to get back to sleep.
- 3 I wake up several hours earlier than I used to and cannot get back to sleep.

17.
- 0 I don't get more tired than usual.
- 1 I get tired more easily than I used to.
- 2 I get tired from doing almost anything.
- 3 I am too tired to do anything.

18.
- 0 My appetite is no worse than usual.
- 1 My appetite is not as good as it used to be.
- 2 My appetite is much worse now.
- 3 I have no appetite at all anymore.

19.
- 0 I haven't lost much weight, if any, lately.
- 1 I have lost more than five pounds.
- 2 I have lost more than ten pounds.
- 3 I have lost more than fifteen pounds.

20.
- 0 I am no more worried about my health than usual.
- 1 I am worried about physical problems like aches, pains, upset stomach, or constipation.
- 2 I am very worried about physical problems and it's hard to think of much else.
- 3 I am so worried about my physical problems that I cannot think of anything else.

21.
- 0 I have not noticed any recent change in my interest in sex.
- 1 I am less interested in sex than I used to be.
- 2 I have almost no interest in sex.
- 3 I have lost interest in sex completely.

INTERPRETING THE BECK DEPRESSION INVENTORY

Now that you have completed the questionnaire, add up the score for each of the twenty-one questions by counting the number to the right of each question you marked. The highest possible total for the whole test would be sixty-three. This would mean you circled number three on all twenty-one questions. Since the lowest possible score for each question is zero, the lowest possible score for the test would be zero. This would mean you circles zero on each question. You can evaluate your depression according to the Table below.

Total Score	Levels of Depression
1-10	These ups and downs are considered normal
11-16	Mild mood disturbance
17-20	Borderline clinical depression
21-30	Moderate depression
31-40	Severe depression
over 40	Extreme depression

http://www.med.navy.mil/sites/NMCP2/PatientServices/SleepClinicLab/Documents/Beck_Depression_Inventory.pdf

Document S2. PERSONAL EVALUATION – BIS 11 A

Name: ______________________________ ***Date:*** ______________

Directions*:* People differ in the ways they act and think in different situations. This is a test to measure some of the ways in which you act and think. Read each statement and darken the appropriate circle on the right side of the page. Do not spend too much time on any statement. Answer quickly and honestly.

		BIS-11A	Translate into BIS-11	SIMILAR	DIFFERENT
1.	I plan tasks carefully …………………………………………	NP	NP	X	
2.	I do things without thinking ………………………………..	M	M	X	
3.	I am happy-go-lucky ………………………………..………..	NP	M		X
4.	I have “racing” thoughts …………………………………….	C/A	C/A	X	
5.	I plan trips well ahead of time …………………………….	NP	NP	X	
6.	I am self-controlled …………………………………………..	M	NP		X
7.	I concentrate easily …………………………………………..	C/A	C/A	X	
8.	I save regularly …………………………………………..……..	NP	NP		
9.	I find it hard to sit still for long periods of time ……………….	M	-		X
10.	I am a careful thinker ……………….………………………..	C/A	NP		X
11.	I plan for job security ………………………………………...	NP	NP	X	
12.	I say things without thinking ……………………………....	M	NP		X
13.	I like to think about complex problems ………………………..	C/A	NP		X
14.	I change jobs …………………………………………………...	NP	M		X
15.	I act “on impulse” ……………………………………………..	M	M		
16.	I get easily bored when solving thought problems ……………..	C/A	NP		X
17.	I have regular medical/dental checkups ………………………	NP	-		X
18.	I act on the spur of the moment ………………………………	M	M	X	
19.	I am a steady thinker …………………………………………..	C/A	C/A	X	
20.	I change where I live …………………………………………..	NP	M		X
21.	I buy things on impulse ………………………………………	M	M	X	
22.	I finish what I start ……………………………………………..	NP	-		X
23.	I walk and move fast …………………………………………....	M	-		X
24.	I solve problems by trial-and-error ……………………………..	C/A	-		X
25.	I spend or charge more than I earn ……………………………..	NP	M		X
26.	I talk fast ………………………………………………………	M	-		X
27.	I have outside thoughts when thinking ………………………..	C/A	C/A	X	
28.	I am more interested in the present than the future ……………..	NP	NP	X	
29.	I am restless at lectures or talks ………………………………	M	C/A		X
30.	I plan for the future ……………………………………………..	NP	M		X

Overlap between BIS-11A and BIS-11 items. The colors represent the original scoring key and the key to translate BIS-11A items into BIS-11 items (green = NP; yellow = C/A; red = M; empty (-) = not present in BIS-11).

BIS 11 $_A$ TO BIS-11: SCORING

Name: ______________________________ ***Date:*** ______________

Directions*:* People differ in the ways they act and think in different situations. This is a test to measure some of the ways in which you act and think. Read each statement and darken the appropriate circle on the right side of the page. Do not spend too much time on any statement. Answer quickly and honestly.

		RARELY/NEVER	OCCASIONALLY	OFTEN	ALMOST ALWAYS / ALWAYS
1.	I plan tasks carefully ..…………………………………………….	**4**	**3**	**2**	**1**
2.	I do things without thinking ……………………………………..	**1**	**2**	**2**	**4**
3.	I am happy-go-lucky ……………………………..…………	**1**	**2**	**2**	**4**
4.	I have “racing” thoughts ……………………………………….	**1**	**2**	**2**	**4**
5.	I plan trips well ahead of time …………………………………	**4**	**3**	**2**	**1**
6.	I am self-controlled ……………………………………………..	**4**	**3**	**2**	**1**
7.	I concentrate easily ……………………………………………..	**4**	**3**	**2**	**1**
8.	I save regularly ……………………………………..……..	**4**	**3**	**2**	**1**
10.	I am a careful thinker ………………..……………………….	**4**	**3**	**2**	**1**
11.	I plan for job security …………………………………………...	**4**	**3**	**2**	**1**
12.	I say things without thinking …………………………………...	**1**	**2**	**2**	**4**
13.	I like to think about complex problems …………………………	**4**	**3**	**2**	**1**
14.	I change jobs …………………………………………………...	**1**	**2**	**2**	**4**
15.	I act “on impulse” ……………………………………………….	**1**	**2**	**2**	**4**
16.	I get easily bored when solving thought problems ……………..	**1**	**2**	**2**	**4**
18.	I act on the spur of the moment …………………………………	**1**	**2**	**2**	**4**
19.	I am a steady thinker ……………………………………………..	**4**	**3**	**2**	**1**
20.	I change where I live ……………………………………………..	**1**	**2**	**2**	**4**
21.	I buy things on impulse …………………………………………	**1**	**2**	**2**	**4**
25.	I spend or charge more than I earn ……………………………..	**1**	**2**	**2**	**4**
27.	I have outside thoughts when thinking …………………………	**1**	**2**	**2**	**4**
28.	I am more interested in the present than the future ……………..	**1**	**2**	**2**	**4**
29.	I am restless at lectures or talks …………………………………	**1**	**2**	**2**	**4**
30.	I plan for the future ……………………………………………..	**4**	**3**	**2**	**1**

Prorating score to BIS-11 Total Impulsiveness: (score/24)*30

BIS 11 $_A$ TO BIS-11: NON-PLANNING IMPULSIVENESS

Name: ______________________________ ***Date:*** ______________

Directions*:* People differ in the ways they act and think in different situations. This is a test to measure some of the ways in which you act and think. Read each statement and darken the appropriate circle on the right side of the page. Do not spend too much time on any statement. Answer quickly and honestly.

		RARELY/NEVER	OCCASIONALLY	OFTEN	ALMOST ALWAYS / ALWAYS
1.	I plan tasks carefully ..………………………………………..	**4**	**3**	**2**	**1**
5.	I plan trips well ahead of time …………………………………	**4**	**3**	**2**	**1**
6.	I am self-controlled ……………………………………………..	**4**	**3**	**2**	**1**
8.	I save regularly …………………………………….…….……..	**4**	**3**	**2**	**1**
10.	I am a careful thinker ………………..………………………	**4**	**3**	**2**	**1**
11.	I plan for job security ………………………………………...	**4**	**3**	**2**	**1**
12.	I say things without thinking ………………………………...	**1**	**2**	**2**	**4**
13.	I like to think about complex problems …………………………	**4**	**3**	**2**	**1**
16.	I get easily bored when solving thought problems ……………..	**1**	**2**	**2**	**4**
28.	I am more interested in the present than the future ……………..	**1**	**2**	**2**	**4**

Prorating score to BIS-11 Non-planning Impulsiveness: (score/10)*11

BIS 11 A TO BIS-11: COGNITIVE/ATTENTIONAL IMPULSIVENESS

Name: ______________________________ ***Date:*** ______________

Directions*:* People differ in the ways they act and think in different situations. This is a test to measure some of the ways in which you act and think. Read each statement and darken the appropriate circle on the right side of the page. Do not spend too much time on any statement. Answer quickly and honestly.

		RARELY/NEVER	OCCASIONALLY	OFTEN	ALMOST ALWAYS / ALWAYS
4.	I have "racing" thoughts ………………………………………….	**1**	**2**	**2**	**4**
7.	I concentrate easily ……………………………………………….	**4**	**3**	**2**	**1**
19.	I am a steady thinker …………………………………………….	**4**	**3**	**2**	**1**
27.	I have outside thoughts when thinking …………………………	**1**	**2**	**2**	**4**
29.	I am restless at lectures or talks …………………………………	**1**	**2**	**2**	**4**

Prorating score to BIS-11 Cognitive Impulsiveness: (score/5)*8

BIS 11 A TO BIS-11: MOTOR IMPULSIVENESS

Name: ______________________________ ***Date:*** ______________

Directions*:* People differ in the ways they act and think in different situations. This is a test to measure some of the ways in which you act and think. Read each statement and darken the appropriate circle on the right side of the page. Do not spend too much time on any statement. Answer quickly and honestly.

		RARELY/NEVER	OCCASIONALLY	OFTEN	ALMOST ALWAYS / ALWAYS
2.	I do things without thinking ……………………………………..	**1**	**2**	**2**	**4**
3.	I am happy-go-lucky ……………………………..…………	**1**	**2**	**2**	**4**
14.	I change jobs ………………………………………………....	**1**	**2**	**2**	**4**
15.	I act “on impulse” ……………………………………………….	**1**	**2**	**2**	**4**
18.	I act on the spur of the moment …………………………………	**1**	**2**	**2**	**4**
20.	I change where I live …………………………………………..	**1**	**2**	**2**	**4**
21.	I buy things on impulse …………………………………………	**1**	**2**	**2**	**4**
25.	I spend or charge more than I earn ……………………………..	**1**	**2**	**2**	**4**
30.	I plan for the future …………………………………………..	**4**	**3**	**2**	**1**

Prorating score to BIS-11 Motor Impulsiveness: (score/9)*11

Document S3. Hospital Anxiety and Depression Scale (HADS)

Tick the box beside the reply that is closest to how you have been feeling in the past week.
Don’t take too long over you replies: your immediate is best.

D	A		D	A	
		**I feel tense or 'wound up':**			**I feel as if I am slowed down:**
	3	Most of the time	3		Nearly all the time
	2	A lot of the time	2		Very often
	1	From time to time, occasionally	1		Sometimes
	0	Not at all	0		Not at all
		**I still enjoy the things I used to enjoy:**			**I get a sort of frightened feeling like 'butterflies' in the stomach:**
0		Definitely as much		0	Not at all
1		Not quite so much		1	Occasionally
2		Only a little		2	Quite Often
3		Hardly at all		3	Very Often
		**I get a sort of frightened feeling as if something awful is about to happen:**			**I have lost interest in my appearance:**
	3	Very definitely and quite badly	3		Definitely
	2	Yes, but not too badly	2		I don't take as much care as I should
	1	A little, but it doesn't worry me	1		I may not take quite as much care
	0	Not at all	0		I take just as much care as ever
		**I can laugh and see the funny side of things:**			**I feel restless as I have to be on the move:**
0		As much as I always could		3	Very much indeed
1		Not quite so much now		2	Quite a lot
2		Definitely not so much now		1	Not very much
3		Not at all		0	Not at all
		**Worrying thoughts go through my mind:**			**I look forward with enjoyment to things:**
	3	A great deal of the time	0		As much as I ever did
	2	A lot of the time	1		Rather less than I used to
	1	From time to time, but not too often	2		Definitely less than I used to
	0	Only occasionally	3		Hardly at all
		**I feel cheerful:**			**I get sudden feelings of panic:**
3		Not at all		3	Very often indeed
2		Not often		2	Quite often
1		Sometimes		1	Not very often
0		Most of the time		0	Not at all
		**I can sit at ease and feel relaxed:**			**I can enjoy a good book or radio or TV program:**
	0	Definitely	0		Often
	1	Usually	1		Sometimes
	2	Not Often	2		Not often
	3	Not at all	3		Very seldom

Please check you have answered all the questions

Scoring:

Total score: Depression (D) ___________ Anxiety (A) ______________

0-7 = Normal

8-10 = Borderline abnormal (borderline case)

11-21 = Abnormal (case)
